# Supplementary material for: Phage-encoded TelN inhibits bacterial Mre11-Rad50 nuclease to protect hairpin telomeres
Source: EMBO J. 2025 Oct 15;44(22):6649–74. doi: 10.1038/s44318-025-00593-z (PMC12624138; doi:10.1038/s44318-025-00593-z)
Supplement: Supplementary file 5 — Expanded View Figures [file 44318_2025_593_MOESM5_ESM.pdf]

Expanded View Figures

Figure EV1. Chromosome linearization in *B. subtilis.*

(**A**) Schematic model for *E. coli* MR sensing and processing of chemically diverse DNA ends. Rad50 comprises an ATP-regulated catalytic head harboring DNA-binding activities, two long, protruding antiparallel coiled coils, and an apical zinc-hook dimerization domain. The DNA-binding and processing head module is formed by the Mre11 nuclease dimer and the Nucleotide-Binding Domains (NBDs) of Rad50. In the resting state, the complex adopts an auto-inhibited conformation where the Rad50 ATPase dimer blocks the Mre11 nuclease active site. Upon DNA binding, the complex transitions to a hypothetical scanning state where it searches for DNA ends along the double helix. Detection of a DNA end triggers an ATP-regulated conformational change, forming the cutting state via a ring-to-rod transition, closing the coiled coils onto a single DNA double helix, repositioning the Mre11 dimer to the side of the complex. MR discriminates against circular DNA, as the presence of two DNA strands within the MR ring prevents the closure of the Rad50 coiled coils. (**B**) *B. subtilis* transformation efficiency as in Fig. 1B but without normalization. (**C**) Suppressor mutations in the specR–*ntelRL* locus in *B. subtilis*. To test whether the *B. subtilis* chromosome can be linearized, we co-introduced *telN* and its cognate *ntelRL* site. No clones carrying both intact *telN* and *ntelRL* were recovered in otherwise wild-type cells; however, rare suppressor clones emerged carrying mutations in or near *ntelRL*. Two-point mutants (Mutants 1 and 2) contain substitutions within the *ntelRL* site itself (red boxes). Two additional suppressor clones (Mutants 3 and 4) contain larger deletions (511 and 225 bp, respectively) spanning the specR–*ntelRL* locus. (**D**) Schematic representation of the chromosomal region analyzed by Southern blotting. The upper panel shows the wild-type (WT) configuration with expected fragments: 4884 bp between BsaI and XhoI (black arrows) and 6755 bp between the two BsaI sites in case of partial XhoI digestion (red dashed arrows). The position of the probe used for hybridization is indicated by a black bar targeting the *ntelRL*-spectinomycin region. The lower panel shows the expected outcome after chromosome linearization. Due to partial XhoI digestion observed in our experiments, additional BsaI-BsaI fragments emerge (red dashed arrows): 7668 bp in the circular context. (**E**) Southern blot analysis of BsaI/XhoI-digested genomic DNA from different *B. subtilis* strains. While our analysis primarily focuses on BsaI-XhoI-digested fragments, partial XhoI digestion resulted in additional bands (marked with red asterisks) representing BsaI-BsaI fragments. Lane 1: WT strain. Lanes 2, 3, and 4: Strains lacking the *B. subtilis* MR complex (ΔMR) and containing the *ntelRL* site inserted adjacent to the spectinomycin resistance gene (specR). Lane 2 (ΔMR + specR_*ntelRL* + P_{xyl}-*telN*) and lane 4 (ΔMR + specR_*ntelRL* + P_{rpsB}-*telN*). An additional 3492 bp band (*) appears due to partial XhoI digestion in these linearized chromosomes. Lane 3 (ΔMR + specR_*ntelRL* + P_{rpsB}-*gfp*): Strain containing the *ntelRL* insertion shows a 5797 bp BsaI-XhoI band and a 7668 bp band (*) from partial XhoI digestion, demonstrating that chromosome linearization specifically requires TelN expression. (**F**) Schematic of pLIN_*ntelN* linear plasmid (12.3 kb) with its DNA hairpin ends (*ntelL* and *ntelR*) depicted in the blowout, adapted from (Liu et al, 2022).

© The Author(s)

A

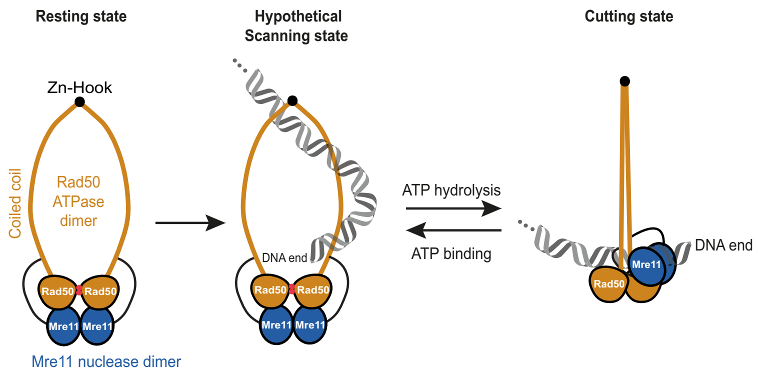

B

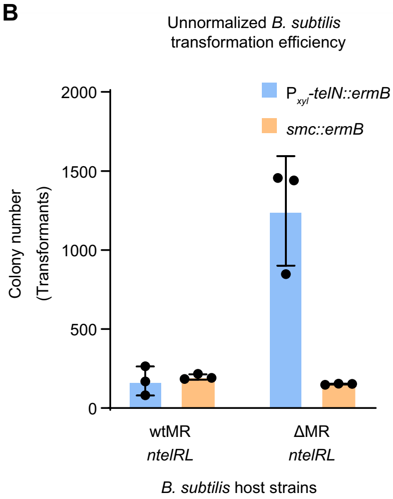

C

B. subtilis suppressor mutations in the *specR–ntelRL* locus

Mutant	Mutation Type	Location	Nucleotide Substitution / Deletion
Mutant 1	Substitution	*ntelRL* site	C19T
Mutant 2	Substitution	*ntelRL* site	T15A, C17T, C19A
Mutant 3	Deletion	*specR–ntelRL* locus	511 bp deletion
Mutant 4	Deletion	*specR–ntelRL* locus	225 bp deletion

1 *ntelR* *ntelL* 56

ntelRL site TATCAGCACACAATTGCCCATTATACGCGCGTATAATGGACTATTGTGTGCTGATA

Mutant 1 TATCAGCACACAATTGCCTATTATACGCGCGTATAATGGACTATTGTGTGCTGATA

Mutant 2 TATCAGCACACAATAGTCAATTATACGCGCGTATAATTGACTATTGTGTGCTGATA

D

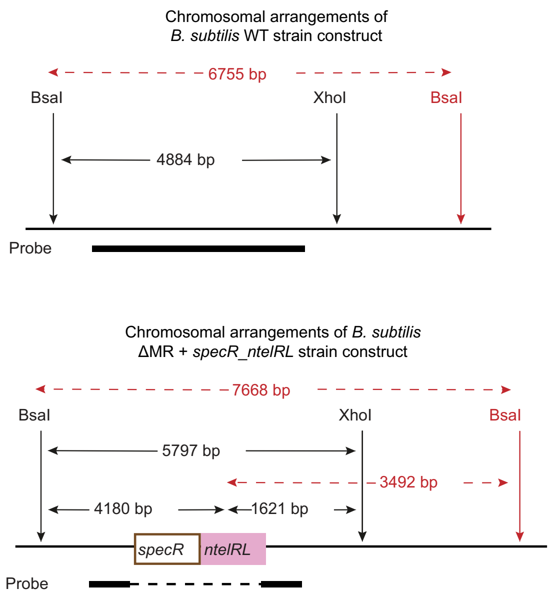

E

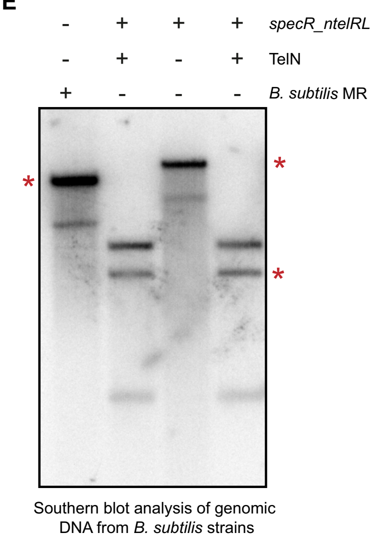

F

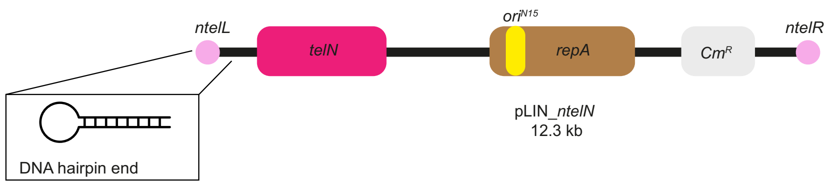

 © The Author(s)

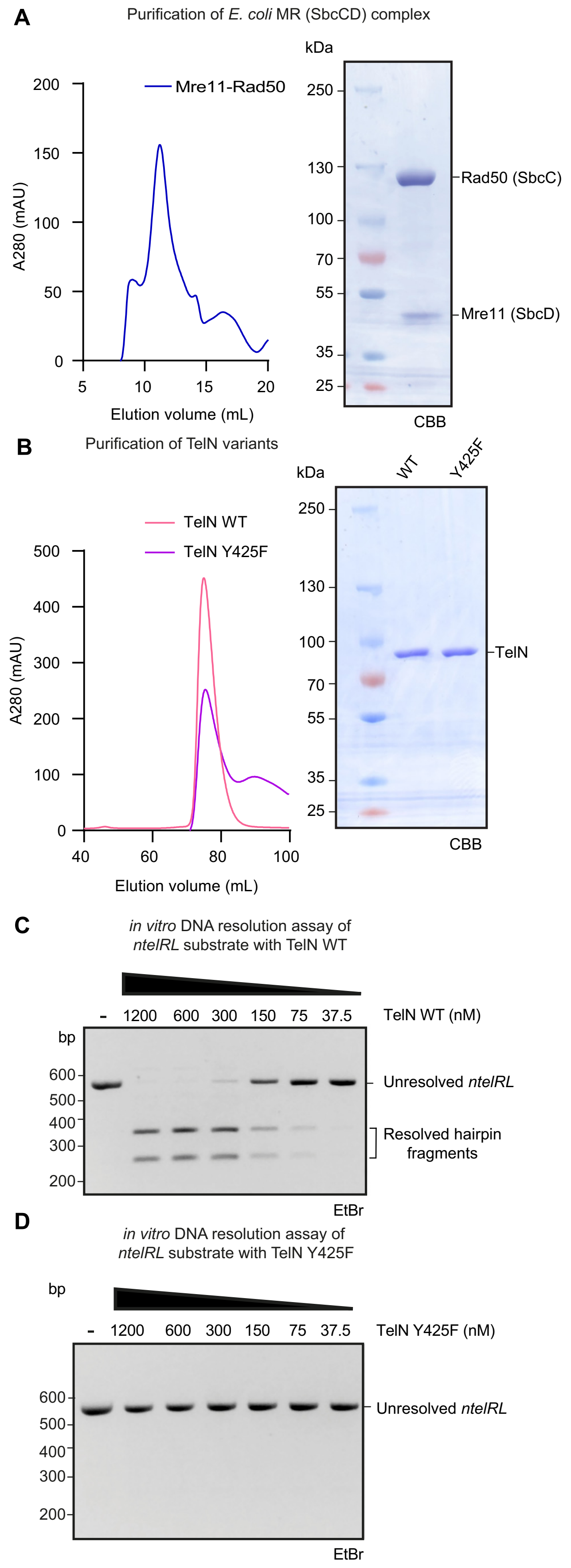
A
Purification of E. coli MR (SbcCD) complex
Mre11-Rad50
A280 (mAU)
Elution volume (mL)
kDa
250
130
100
70
55
35
25
Rad50 (SbcC)
Mre11 (SbcD)
CBB
B
Purification of TelN variants
TelN WT
TelN Y425F
WT
Y425F
TelN
C
in vitro DNA resolution assay of ntelRL substrate with TelN WT
- 1200 600 300 150 75 37.5
TelN WT (nM)
bp
Unresolved ntelRL
Resolved hairpin fragments
EtBr
D
in vitro DNA resolution assay of ntelRL substrate with TelN Y425F
TelN Y425F (nM)

Figure EV2. Protein purification and characterization.

(**A**, **B**) Left panels: representative size-exclusion chromatography (SEC) elution profile for the purification of (**A**) the *E. coli* MR complex, (**B**) TelN WT and TelN(Y425F) proteins. Right panels: SDS-PAGE profile of purified fractions of (**A**) MR, (**B**) TelN WT, and TelN(Y425F) as visualized by Coomassie brilliant blue (CBB) staining. (**C**, **D**) DNA resolution assays on *ntelRL* DNA substrate (*ntelRL* with additional neighboring sequences from N15 phage) using decreasing concentrations of (**C**) TelN WT and (**D**) TelN(Y425F). DNA species were resolved with a 1.5% ethidium bromide (EtBr) agarose gel.

 © The Author(s)

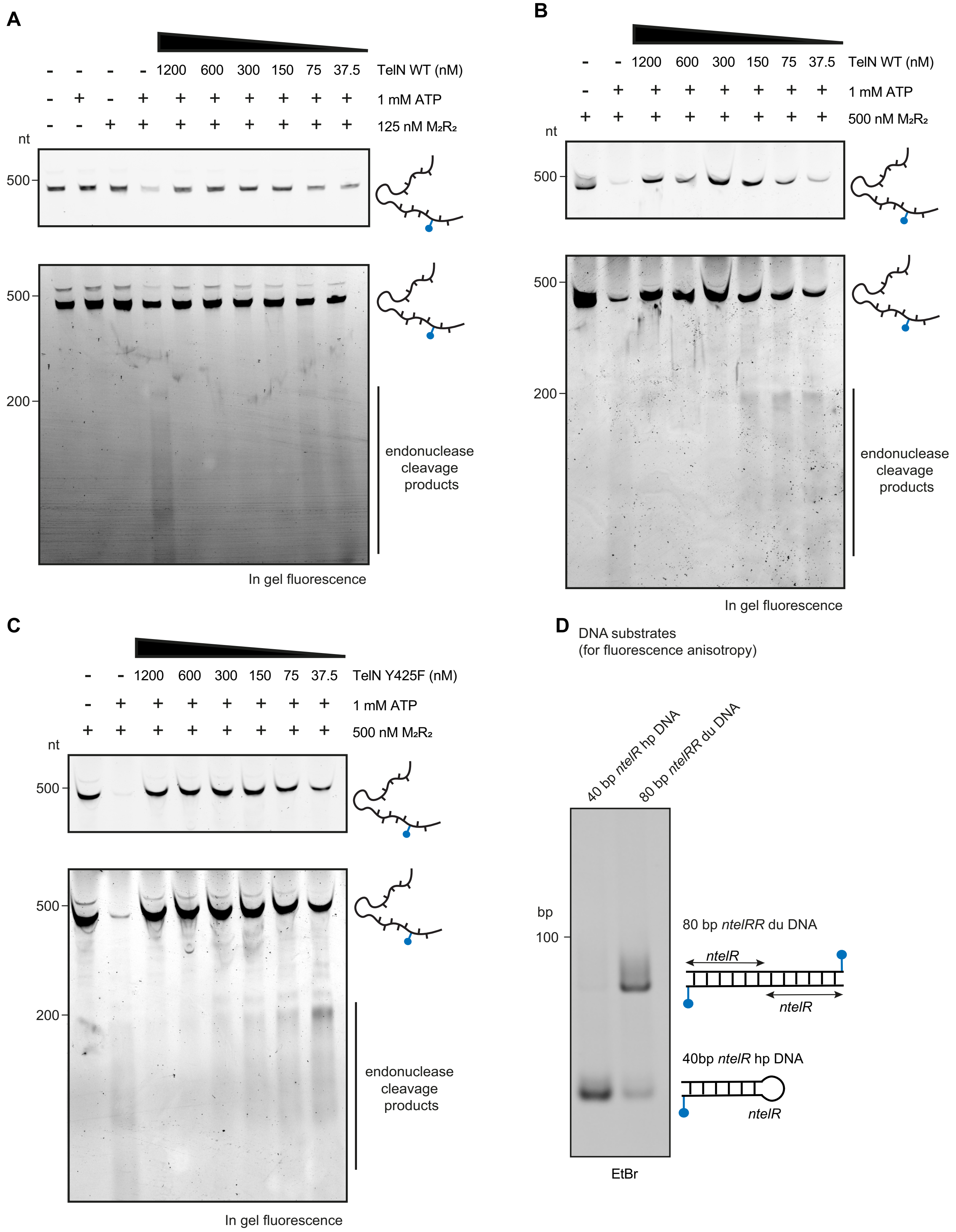
A
- - - - 1200 600 300 150 75 37.5 TelN WT (nM)
- + - + + + + + + + 1 mM ATP
- - + + + + + + + + 125 nM M2R2
nt
500
500
200
endonuclease cleavage products
In gel fluorescence
B
- - 1200 600 300 150 75 37.5 TelN WT (nM)
- + + + + + + + 1 mM ATP
+ + + + + + + + 500 nM M2R2
nt
500
500
200
endonuclease cleavage products
In gel fluorescence
C
- - 1200 600 300 150 75 37.5 TelN Y425F (nM)
- + + + + + + + 1 mM ATP
+ + + + + + + + 500 nM M2R2
nt
500
500
200
endonuclease cleavage products
In gel fluorescence
D
DNA substrates
(for fluorescence anisotropy)
40 bp ntelR hp DNA
80 bp ntelRR du DNA
bp
100
80 bp ntelRR du DNA
ntelR
ntelR
40bp ntelR hp DNA
ntelR
EtBr

© The Author(s)

Figure EV3. TelN DNA binding and protection assays.

(**A–C**) In vitro DNA protection assays using (**A**) TelN WT with 125 nM M_2R_2 (including ATP-only control), (**B**) TelN WT with 500 nM M_2R_2, or (**C**) TelN(Y425F) with 500 nM M_2R_2. About 37 nM of the immobilized *ntelL* hairpin substrate (237 bp) was incubated with the respective M_2R_2 concentration and decreasing concentrations of the indicated TelN variant. Products were analyzed by DNA-denaturing PAGE after biotin/SDS elution. Lower panel: representative in-gel fluorescence analysis showing DNA degradation profile. Upper panel: same gel image with exposure optimized to visualize remaining substrate bands. (**D**) Analysis of fluorescently labeled DNA substrates on a 10% native polyacrylamide gel. The substrates used for binding measurements with TelN WT and TelN(Y425F) include a 40 bp *ntelR* hairpin (hp) DNA and an 80 bp *ntelRR* duplex (du) DNA. Both substrates were derived from the same 80-nucleotide palindromic oligonucleotide, which contains the *ntelR* sequence followed by its reverse complement. The palindromic nature of this sequence allows it to form either a 40 bp hairpin through intramolecular annealing or an 80 bp dsDNA through intermolecular annealing, depending on preparation conditions.

 © The Author(s)

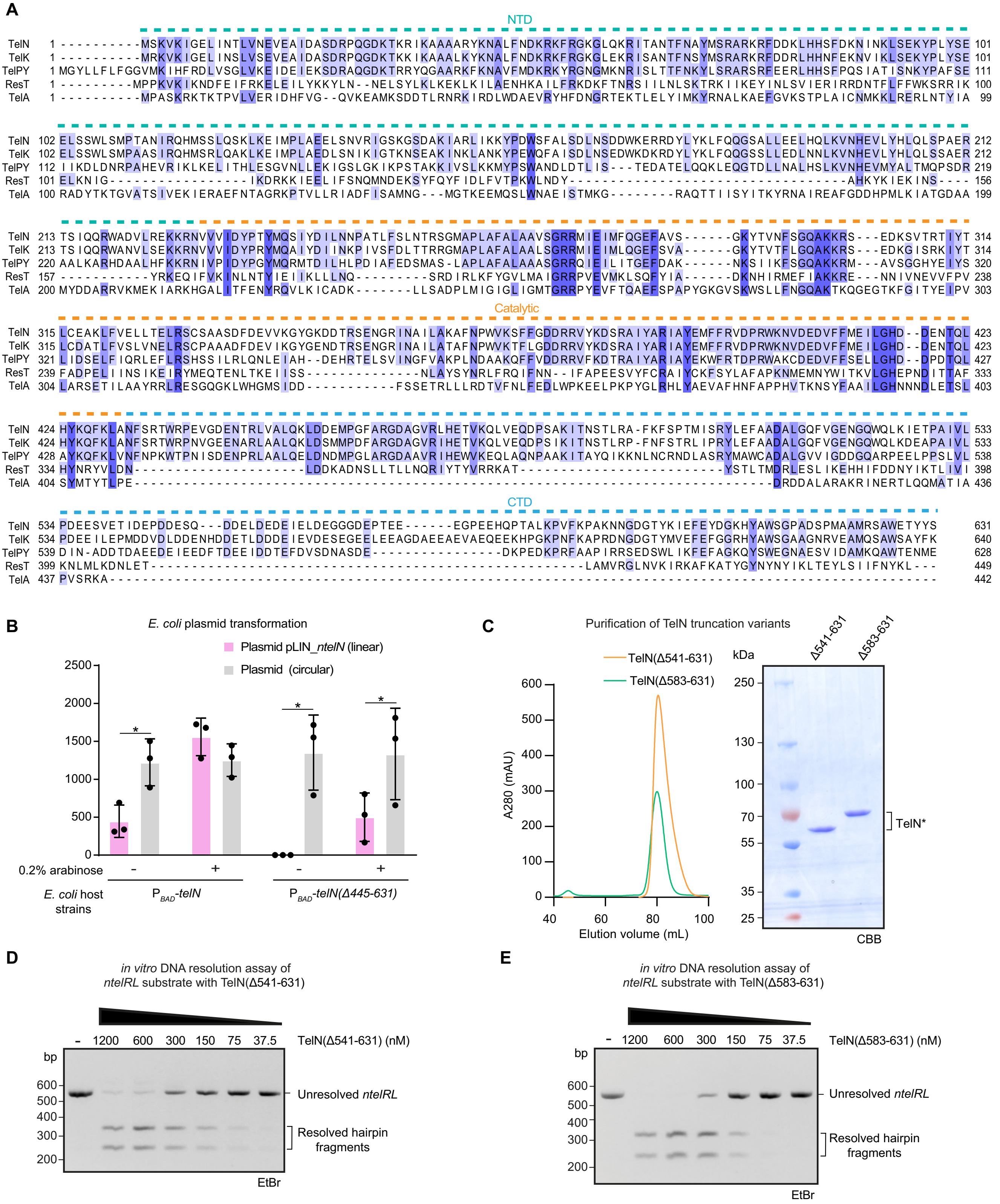
A
NTD
TelN 1 ----------MSKVKIGELINTLVNEVEAIDASDRPQGDKTKRIKAAAARYKNALFNDKRKFRGKGLQKRITANTFNAYMSRARKRFDDKLHHSFDKNINKLSEKYPLYSE 101
TelK 1 ----------MRKVKIGELINSLVSEVEAIDASDRPQGDKTKKIKAAALKYKNALFNDKRKFRGKGLEKRISANTFNSYMSRARKRFDDRLHHNFEKNVIKLSEKYPLYSE 101
TelPY 1 MGYLLFLFGGVMKIHFRDLVSGLVKEIDEIEKSDRAQGDKTRRYQGAARKFKNAVFMDKRKYRGNGMKNRISLTTFNKYLSRARSRFEERLHHSFPQSIATISNKYPAFSE 111
ResT 1 ---------MPPKVKIKNDFEIFRKELEILYKKYLN--NELSYLKLKEKLKILAENHKAILFRKDKFTNRSIILNLSKTRKIIKEYINLSVIERIRRDNTFLFFWKSRRIK 100
TelA 1 ----------MPASKRKTKTPVLVERIDHFVG--QVKEAMKSDDTLRNRKIRDLWDAEVRYHFDNGRTEKTLELYIMKYRNALKAEFGVKSTPLAICNMKKLRERLNTYIA 99
TelN 102 ELSSWLSMPTANIRQHMSSLQSKLKEIMPLAEELSNVRIGSKGSDAKIARLIKKYPDWSFALSDLNSDDWKERRDYLYKLFQQGSALLEELHQLKVNHEVLYHLQLSPAER 212
TelK 102 ELSSWLSMPAASIRQHMSRLQAKLKEIMPLAEDLSNIKIGTKNSEAKINKLANKYPEWQFAISDLNSEDWKDKRDYLYKLFQQGSSLLEDLNNLKVNHEVLYHLQLSSAER 212
TelPY 112 IIKDLDNRPAHEVRIKLKELITHLESGVNLLEKIGSLGKIKPSTAKKIVSLKKMYPSWANDLDTLIS---TEDATELQQKLEQGTDLLNALHSLKVNHEVMYALTMQPSDR 219
ResT 101 ELKNIG-------------------------------------IKDRKKIEELIFSNQMNDEKSYFQYFIDLFVTPKWLNDY--------------------------AHKYKIEKINS---- 156
TelA 100 RADYTKTGVATSIVEKIERAEFNTAGRKPTVLLRIADFISAMNG---MGTKEEMQSLWNAEISTMKG--------RAQTTIISYITKYRNAIREAFGDDHPMLKIATGDAA 199
TelN 213 TSIQQRWADVLREKKRNVVVIDYPTYMQSIYDILNNPATLFSLNTRSGMAPLAFALAAVSGRRMIEIMFQGEFAVS------GKYTVNFSGQAKKRS---EDKSVTRTIYT 314
TelK 213 TSIQQRWANVLSEKKRNVVVIDYPRYMQAIYDIINKPIVSFDLTTRRGMAPLAFALAALSGRRMIEIMLQGEFSVA------GKYTVTFLGQAKKRS---EDKGISRKIYT 314
TelPY 220 AALKARHDAALHFKKRNIVPIDYPGYMQRMTDILHLPDIAFEDSMAS-LAPLAFALAAASGRRQIEILITGEFDAK------NKSIIKFSGQAKKRM---AVSGGHYEIYS 320
ResT 157 -----------YRKEQIFVKINLNTYIEIIKLLLNQ---------SRDIRLKFYGVLMAIGRRPVEVMKLSQFYIA------DKNHIRMEFIAKKRE---NNIVNEVVFPV 238
TelA 200 MYDDARRVKMEKIARKHGALITFENYRQVLKICADK-------LLSADPLMIGIGLIGMTGRRPYEVFTQAEFSPAPYGKGVSKWSLLFNGQAKTKQGEGTKFGITYEIPV 303
Catalytic
TelN 315 LCEAKLFVELLTELRSCSAASDFDEVVKGYGKDDTRSENGRINAILAKAFNPWVKSFFGDDRRVYKDSRAIYARIAYEMFFRVDPRWKNVDEDVFFMEILGHD--DENTQL 423
TelK 315 LCDATLFVSLVNELRSCPAAADFDEVIKGYGENDTRSENGRINAILATAFNPWVKTFLGDDRRVYKDSRAIYARIAYEMFFRVDPRWKNVDEDVFFMEILGHD--DENTQL 423
TelPY 321 LIDSELFIQRLEFLRSHSSILRLQNLEIAH--DEHRTELSVINGFVAKPLNDAAKQFFVDDRRVFKDTRAIYARIAYEKWFRTDPRWAKCDEDVFFSELLGHD--DPDTQL 427
ResT 239 FADPELIINSIKEIRYMEQTENLTKEIISS--------------NLAYSYNRLFRQIFNN--IFAPEESVYFCRAIYCKFSYLAFAPKNMEMNYWITKVLGHEPNDITTAF 333
TelA 304 LARSETILAAYRRLRESGQGKLWHGMSIDD-----------FSSETRLLRDTVFNLFEDLWPKEELPKPYGLRHLYAEVAFHNFAPPHVTKNSYFAAILGHNNNDLETSL 403
TelN 424 HYKQFKLANFSRTWRPEVGDENTRLVALQKLDDEMPGFARGDAGVRLHETVKQLVEQDPSAKITNSTLRA-FKFSPTMISRYLEFAADALGQFVGENGQWQLKIETPAIVL 533
TelK 424 HYKQFKLANFSRTWRPNVGEENARLAALQKLDSMMPDFARGDAGVRIHETVKQLVEQDPSIKITNSTLRP-FNFSTRLIPRYLEFAADALGQFVGENGQWQLKDEAPAIVL 533
TelPY 428 AYKQFKLVNFNPKWTPNISDENPRLAALQELDNDMPGLARGDAAVRIHEWVKEQLAQNPAAKITAYQIKKNLNCRNDLASRYMAWCADALGVVIGDDGQARPEELPPSLVL 538
ResT 334 HYNRYVLDN-------------------LDDKADNSLLTLLNQRIYTYVRRKAT-----------------------YSTLTMDRLESLIKEHHIFDDNYIKTLIVI 398
TelA 404 SYMTYTLPE---DRDDALARAKRINERTLQQMATIA 436
CTD
TelN 534 PDEESVETIDEPDDESQ---DDELDEDEIELDEGGGDEPTEE------EGPEEHQPTALKPVFKPAKNNGDGTYKIEFEYDGKHYAWSGPADSPMAAMRSAWETYYS 631
TelK 534 PDEEILEPMDDVDLDDENHDDETLDDDEIEVDESEGEELEEAGDAEEAEVAEQEEKHPGKPNFKAPRDNGDGTYMVEFEFGGRHYAWSGAAGNRVEAMQSAWSAYFK 640
TelPY 539 DIN-ADDTDAEEDEIEEDFTDEEIDDTEFDVSDNASDE---------------DKPEDKPRFAAPIRRSEDSWLIKFEFAGKQYSWEGNAESVIDAMKQAWTENME 628
ResT 399 KNLMLKDNLET--LAMVRGLNVKIRKAFKATYGYNYNYIKLTEYLSIIFNYKL--- 449
TelA 437 PVSRKA--- 442
B
E. coli plasmid transformation
Plasmid pLIN_ntelN (linear)
Plasmid (circular)
2500
2000
1500
1000
500
0
*
*
*
0.2% arabinose
-
+
-
+
E. coli host strains
PBAD-telN
PBAD-telN(Δ445-631)
C
Purification of TelN truncation variants
TelN(Δ541-631)
TelN(Δ583-631)
A280 (mAU)
600
500
400
300
200
100
0
40
60
80
100
Elution volume (mL)
kDa
Δ541-631
Δ583-631
250
130
100
70
55
35
25
TelN*
CBB
D
in vitro DNA resolution assay of ntelRL substrate with TelN(Δ541-631)
-
1200
600
300
150
75
37.5
TelN(Δ541-631) (nM)
bp
600
500
400
300
200
Unresolved ntelRL
Resolved hairpin fragments
EtBr
E
in vitro DNA resolution assay of ntelRL substrate with TelN(Δ583-631)
-
1200
600
300
150
75
37.5
TelN(Δ583-631) (nM)
bp
600
500
400
300
200
Unresolved ntelRL
Resolved hairpin fragments
EtBr

Figure EV4. Design and characterization of TelN truncation mutants.

(**A**) Multiple-sequence alignment of telomere resolvases, including TelN from *E. coli* phage N15, TelK from *Klebsiella oxytoca* phage phiKO2, TelPY from *Yersinia* phage PY54, TelA from *Agrobacterium tumefaciens*, and ResT from *Borrelia burgdorferi*. Numbers indicate amino acid positions. The sequence alignment was done on JalView; residues are color-coded based on percentage identity. TelN domains are indicated by colored dashed lines: N-ter domain (NTD, green dashed line), catalytic domain (orange dashed line), and C-ter domain (CTD, blue dashed line). (Uniprot accession numbers: TelN, Q37967; TelK, Q6UAV6; TelPY, Q7Y3Y3; ResT, O50979; TelA, F8KAE9). (**B**) Counts of chloramphenicol-resistant colonies for the indicated *E. coli* strains after transformation with either pLIN_*ntelN* or a control circular plasmid. P_{BAD}-*telN(Δ445–631)*: strain with chromosomal integration of arabinose-inducible TelN(Δ445–631). Means and standard deviations from three biological replicates are shown. Asterisks indicate samples with a *p* value, obtained by paired *t*-tests, lower than 0.05. P_{BAD}-*telN* without arabinose: $p = 0.0233$; P_{BAD}-*telN(Δ445–631)* without arabinose: $p = 0.0420$; P_{BAD}-*telN(Δ445–631)* with arabinose: $p = 0.0397$. (**C**) Left panel: representative size-exclusion chromatography (SEC) elution profiles for the purification of TelN(Δ541–631) and TelN(Δ583–631) truncation proteins. Right panel: SDS-PAGE profiles of purified proteins visualized by Coomassie brilliant blue (CBB) staining. (**D**, **E**) DNA resolution assays on *ntelRL* DNA substrate using decreasing concentrations of (**D**) TelN(Δ541–631) and (**E**) TelN(Δ583–631). DNA species were resolved on a 1.5% EtBr agarose gel.

© The Author(s)

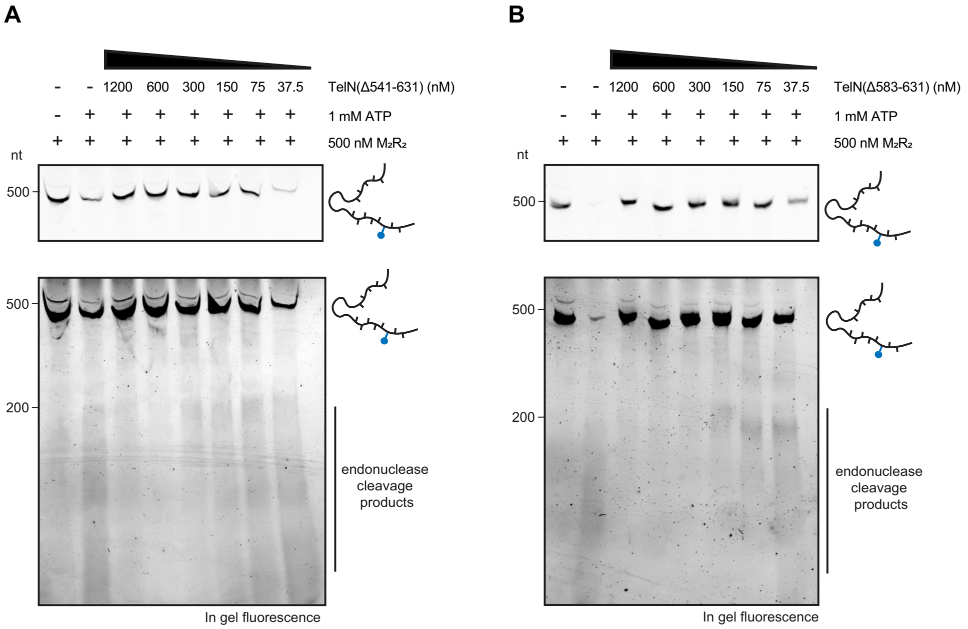

Figure EV5. In vitro DNA protection assays with Mre11-Rad50.

(**A**) TelN(Δ541–631) and (**B**) TelN(Δ583–631). Products were analyzed by DNA-denaturing PAGE after biotin/SDS elution. Lower panel: representative in-gel fluorescence analysis showing DNA degradation profile. Upper panel: same gel image with exposure optimized to visualize remaining substrate bands.

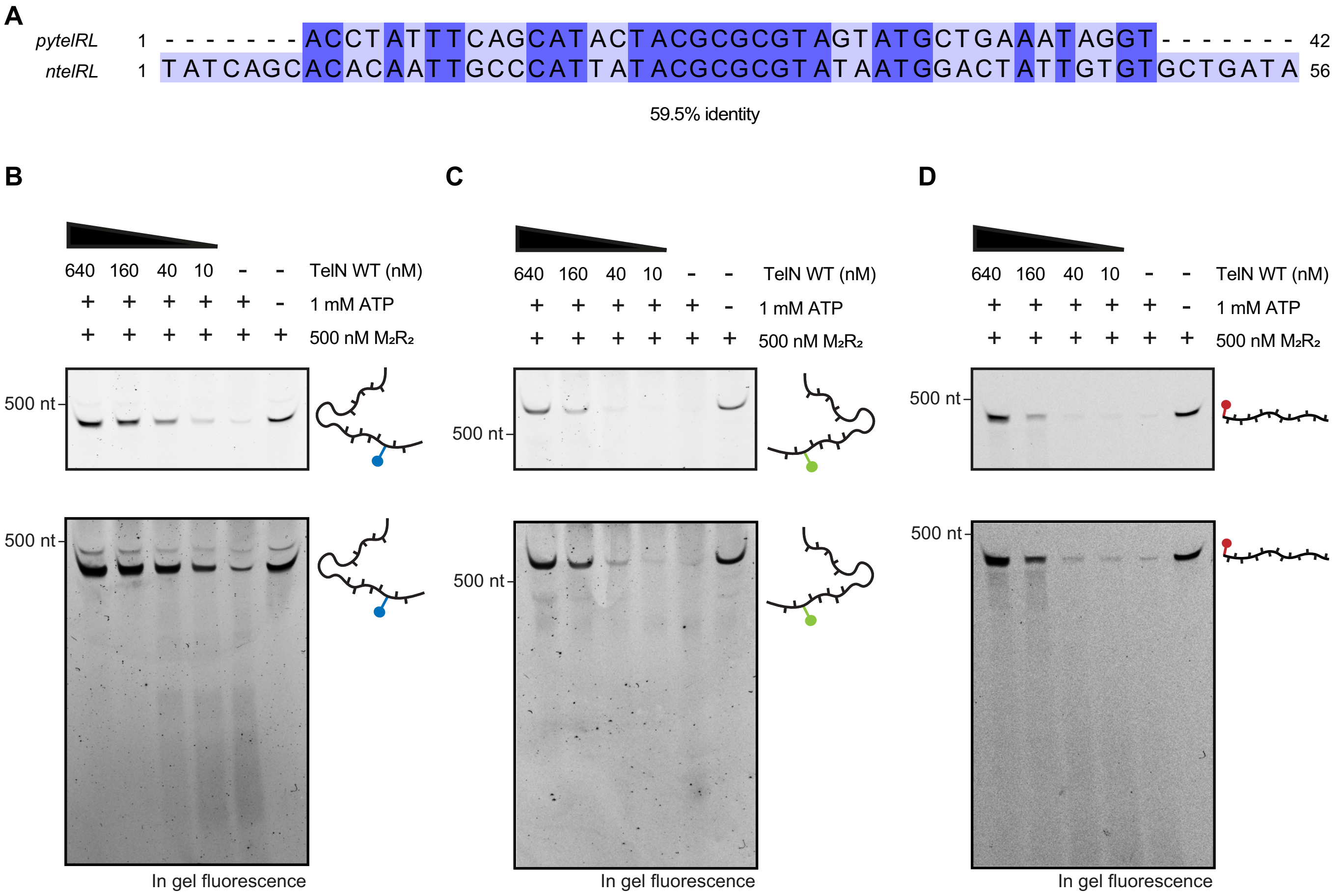

Figure EV6. DNA specificity of DNA protection.

(**A**) Sequence alignment of the recognition sequences *pytelRL* (42 bp) and *ntelRL* (56 bp). Numbers above the sequences indicate nucleotide positions. The alignment was performed using JalView software; nucleotides are color-coded based on percentage identity. (**B**, **D**) Individual gel scans corresponding to the overlay image shown in Fig. 4C for a clearer interpretation of substrate-specific degradation patterns. Lower panels: representative in-gel fluorescence analysis showing DNA degradation profile. Upper panels: same gel image with exposure optimized to visualize remaining substrate bands. The cartoons next to each gel indicate the DNA substrate used in the assay: (**B**) bead-immobilized 237 bp *ntelL* hairpin DNA substrate labeled with blue fluorescence, (**C**) 329 bp *ntelR* hairpin DNA substrate labeled with green fluorescence, and (**D**) 411 bp nonspecific linear DNA substrate labeled with red fluorescence.

 © The Author(s)
